# Green synthesis and characterization of zinc oxide nanoparticles using bush tea ( *Athrixia phylicoides* DC) natural extract: assessment of the synthesis process.

**DOI:** 10.12688/f1000research.73272.4

**Published:** 2022-08-23

**Authors:** Gabriel Amani Kaningini, Shohreh Azizi, Hlengilizwe Nyoni, Fhatuwani Nixwel Mudau, Keletso Cecilia Mohale, Malik Maaza

**Affiliations:** 1 UNESCO-UNISA Africa Chair in Nanosciences and Nanotechnology College of Graduates Studies University of South Africa, Muckleneuk Ridge, Pretoria, 392, South Africa; 2 Nanosciences African Network (NANOAFNET) iThemba LABS-National Research Foundation, 1 Old Faure Road, Somerset West, Western Cape, 7129 PO Box 722, South Africa; 3 Nanotechnology and Water Sustainability Research (NanoWS) Unit, College of Science Engineering and Technology, University of South Africa, Johannesburg, 1709, South Africa; 4 Department of Agriculture and Animal Health, College of Agriculture and Environmental Sciences, University of South Africa, Private Bag X6, Florida, 1710, South Africa

**Keywords:** ZnO nanoparticles, green synthesis, bush tea, reducing agent, natural extract.

## Abstract

**Background: **Nanoparticles are globally synthesized for their antimicrobial, anti-inflammatory, wound healing, catalytic, magnetic, optical, and electronic properties that have put them at the forefront of a wide variety of studies. Among them, zinc oxide (ZnO) has received much consideration due to its technological and medicinal applications. In this study, we report on the synthesis process of ZnO nanoparticles using *Athrixia phylicoides* DC natural extract as a reducing agent.

**Methods:** Liquid chromatography–mass spectrometry (LC-MS) was used to identify the compounds responsible for the synthesis of ZnO nanoparticles. Structural, morphological and optical properties of the synthesized nanoparticles have been characterized through X-ray diffraction (XRD), Ultraviolet-visible spectroscopy (UV-Vis), Fourier transform infrared spectroscopy (FTIR), scanning electron microscopy (SEM) and energy-dispersive X-ray spectroscopy (EDS).

**Results:** LC-MS results showed that different flavonoids and polyphenols, as well as Coumarin, an aromatic compound, reacted with the precursor to form ZnO nanoparticles. XRD and UV-Vis analysis confirmed the synthesis of ZnO nanoparticles, with a spherical shape showed in SEM images. The quasi-spherical ZnO crystals had an average crystallite size of 24 nm. EDS and FTIR analysis confirmed that the powders were pure with no other phase or impurity.

**Conclusions:** This study successfully demonstrated that the natural plant extract of *A. phylicoides* DC. can be used in the bio-reduction of zinc nitrate hexahydrate to prepare pure ZnO nanoparticles, thus, extending the use of this plant to an industrial level.

## Introduction

Nanomaterials with a size less than 100 nm are globally synthesized owing to their various properties such as, antimicrobial, anti-inflammatory, wound healing, catalytic, magnetic, optical, and electronic properties, that have put them at the forefront of a wide variety of studies ( Gunalan *et al*., 2012; Jamdagni *et al*., 2018). Compared to their counterpart bulk materials, they present a reduced surface-to-volume ratio that increases as the size is reduced ( Ohno *et al*., 2010), thus placing them on the borderline between single molecules and bulk materials ( Mansoori and Soelaiman, 2005).

The introduction of nanoparticles in the consumer industry, health, food, space, chemical, and cosmetics, has called for a green and environmentally responsible strategy for their production ( Rao and Gautam, 2016). Metal oxides and dioxides such as zinc oxide, silver, gold and titanium dioxide have received copious consideration because of their multiple properties and applications ( Dobrucka and Długaszewska, 2016). However, their synthesis has been done through numerous physicochemical methods. Laser ablation, microwave irradiation and vapour deposition have been reported to date ( Satyanarayana and Reddy, 2018), they involve forces of condensation, dispersion, or fragmentation of bulk particles into nanoparticles, as well as some toxic chemicals, harmful to the environment ( Dhandapani *et al*., 2014; Krupa and Vimala, 2016).

Synthesis of nanomaterials through biological systems assisted by some biotechnological tools is an emerging asset of nanotechnology that provides a safe, cost-effective and eco-friendly synthesis process ( Shinwari and Maaza, 2017). Plants, diatoms, fungi, yeast, algae, bacteria, and human cells have been used. Their proteins and other metabolites have been well reported to have a reductive capacity that can transform metal ions into metal nanoparticles ( Dobrucka and Długaszewska, 2016; Parveen *et al*., 2016). The biological synthesis of nanoparticles provides more advantages than chemical and physical ones ( Kharissova *et al*., 2013). Numerous metal oxide nanoparticles, such as TiO _2_, CuO, and ZnO have been produced by total green chemistry. Among them, ZnO, an n-type semiconductor, has gained interest owing to its easy production, cost-effectiveness, and safety of synthesis and usage ( Agarwal *et al*., 2017). Several studies have successfully been led to synthesize ZnO nanoparticles using different organisms such as bacteria, fungi, algae, and plants ( Agarwal *et al*., 2017).

Among all biological systems, phytosynthesis of nanoparticles using plants has shown great potential. Plant-mediated nanoparticle synthesis is simple, eco-friendly, and provides antibacterial assets ( Gunalan *et al*., 2012; Iravani *et al*., 2015; Thema *et al*., 2015). A variety of metabolites such as terpenoids, polyphenols, sugars, alkaloids, phenolic acids and proteins have been reported to have metal ion reduction assets ( Parveen *et al*., 2016). Several studies dedicated to the green synthesis of ZnO nanoparticles using plant extracts as capping or reducing agents have shown the use of different plant aerial parts, such as leaves and fruits of different species such as *Aloe vera*, *Hibiscus sabdariffa*, *Allium sativum*, *Allium cepa*, *Petroselinum crispum, Moringa oleifera* and *Camellia sinensis,* for the synthesis of nanoparticles ( Mahendiran *et al*., 2017; Matinise *et al*., 2017; Senthilkumar and Sivakumar, 2014; Stan *et al*., 2015). Bush tea, mostly known as a medicinal tea plant in southern Africa where it originates has high concentrations of phenolic compounds such as tannins and flavonoids ( Lerotholi *et al*., 2017). However, data explaining the synthesis processes of nanoparticles using this plant are lacking. Hence, the objective of this study was to contribute to the explanation of the compounds induced in the synthesis process of ZnO nanoparticles using *Athrixia phylicoides* leaf extract.

## Methods

### Material

Leaves of bush tea ( *A.* *phylicoides* DC) were used to reduce zinc nitrate hexahydrate. Analytical grade Zn (NO _3_) _2_.6H _2_O of 99% purity was purchased from Sigma-Aldrich, South Africa. Bush tea leaves were harvested from the wild in Thohoyandou (22.8785°S; 30.4818°E) in the Limpopo province, South Africa. Following the harvest, the leaves were washed with deionized water and freeze-dried for 72 hours at -50°C at a pressure of 0.32 Kpa, hereafter they were ground and kept for further usage.

### Material preparation

Extract preparation

Ten grams of ground bush tea leaves were weighed and mixed with 300 ml of deionized water. The mixture was heated at 60°C for 30 minutes until the water changed to a dark green colour. After centrifugation using a Hermle Labortecnik GmbH Z 216-M benchmark centrifuge at 4000 rpm for 10 minutes, the mixture was filtered twice using Whatman filter paper number 1, and the extract was kept in an airtight container in a fridge at ≈4°C for analysis and ZnO nanoparticles synthesis.

Synthesis of ZnO nanoparticles

In this study, zinc nitrate hexahydrate [Zn (NO _3_) _2_.6H _2_O] was used as the precursor. One gram of the precursor was mixed with 25 ml of *A. phylicoides* extract. The mixture was kept on a magnetic stirrer at 300 rpm at 60°C for 30 minutes and then left to cool down at room temperature for 12 hours, a precipitate was observed. The mixture was centrifuged for 15 minutes at 4000 rpm. The supernatant was collected and transferred to LC-MS vials for analysis and the precipitate was dried at 60°C for one hour and then annealed at 800°C for two hours. The obtained powder was then kept for characterization.

Bush tea compounds profiling and identification

The determination and profiling of different compounds present in the extract before the synthesis as well as the supernatant after synthesis were performed using liquid chromatography quadrupole time-of-flight mass spectrometry (LC-Q-TOF-MS) using a Bruker impact II (Germany). After peak integration and Pareto scaling, the liquid chromatography-mass spectrometry (LC-MS) data were transformed into buckets using the Bruker Compass data analysis programme version 4.3.110 ( https://www.bruker.com/en/). Peaks were determined using real mass, MS/MS, and retention time (RT). The accuracy of the mass and MS/MS spectral data was compared to the Kyoto standard Encyclopaedia of Genes and Genomes (KEGG) and ChemSpider databases using the MetFrag 2.2 online software ( Tete *et al*., 2020). Principal component analysis (PCA) and T-tests were performed using MetaboAnalyst 4.0.

ZnO nanoparticle characterization

The characterization of the obtained ZnO nanopowders was done using X-ray diffraction (XRD), Ultraviolet-visible spectroscopy (UV-Vis), Fourier-transform infrared spectroscopy (FTIR), scanning electron microscopy (SEM) and energy-dispersive X-ray spectroscopy (EDS). The crystallite size of ZnO nanoparticles was estimated using the modified Scherrer equation:

$$L=\frac{K\lambda }{\beta \text{cosθ}}$$

where λ is the X-ray wavelength, *β* the peak width at half maximum weight, *K* = 0.9, and the Scherrer constant ( Monshi *et al*., 2012).

## Results

### Assessment of the synthesis process

Evaluation of extract composition relative to the synthesis of ZnO nanoparticles using LC-Q-TOF-MS

**Figure 1.**
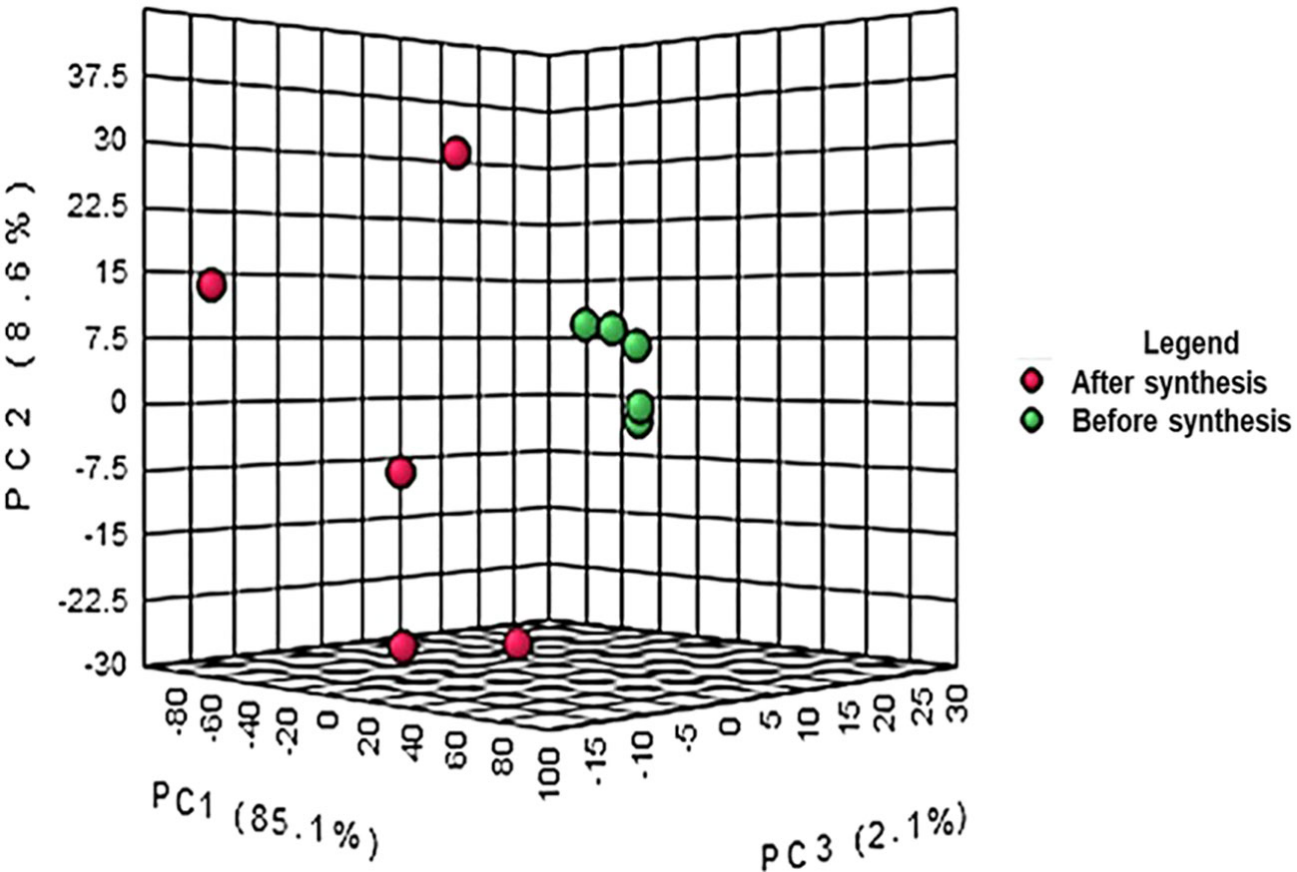
Principal component analysis of liquid chromatography quadrupole time-of-flight mass spectrometry (LC-Q-TOF-MS) peak intensities of 10 bush tea ( *Athrixia phylicoides* DC.) leaf extracts before and after ZnO nanoparticle synthesis.

The crude extract from bush tea leaves and the supernatant after the synthesis of ZnO nanoparticles were investigated. The differences between the composition of the crude extract and the supernatant after synthesis are represented in Figure 1. The results from the PCA co-variance of data show that two distinct groups were observed from the three principal components with 85.1%, 8.6% and 2.1% respectively for principal components 1, 2 and 3. The compounds (represented in red) resulting from the supernatant after synthesis of ZnO nanoparticles clustered together following the Y-axis of the PCA while the compounds of crude extract were on the Z-axis. The differences observed are due to the reaction between the plant extract and the precursor to form ZnO nanoparticles. The synthesis of ZnO nanoparticles involves a reaction between the plant extract and the precursor resulting in the reduction of Zn ^+2^ ions into ZnO nanoparticles ( Hussain *et al*., 2019).

**Figure 2.**
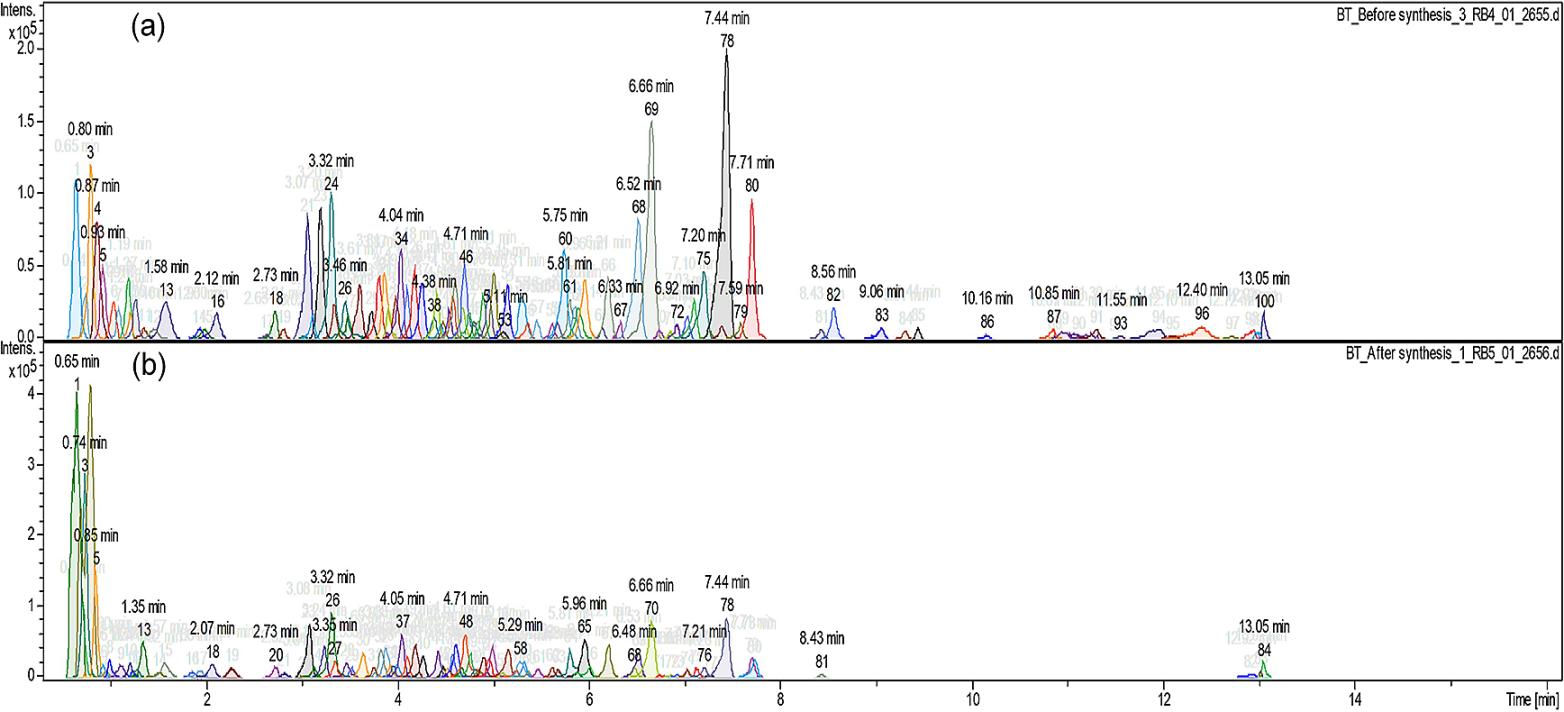
Compound dissection (a) before the synthesis process (100 peaks) (b) after the synthesis process (84 peaks).

Figure 2 shows the different compound peaks observed using LC-Q-TOF-MS analysis. The dissection of observed spectra into compounds produced 100 different peaks for the crude extract ( Figure 2a) and 84 peaks for the supernatant after synthesis ( Figure 2b). The reduction in the number of compounds confirms that the synthesis took place and secondary metabolites from *A.* *phylicoides* DC. The extract has reacted with the precursor reducing Zn ^2+^ ions into ZnO nanoparticles.

Bush tea extract compounds identification before and after synthesis

Different peaks identified, after chromatogram dissection ( Figure 2), from LC-Q-TOF-MS revealed the presence of several compounds in both the crude extract and the supernatant after ZnO nanoparticle synthesis, with a reduction of the compound’s amount in the supernatant collected after synthesis. Thus, revealing the presence of an interaction between the precursor and the extract mainly by the oxidation, reduction or degradation of the phytochemical compounds that occur during nanoparticle formation ( Jeevanandam *et al*., 2016). Table 1 presents the secondary metabolites investigated for both the crude bush tea extract and the supernatant solution after synthesis, respectively.

**Table 1.** Liquid chromatography quadrupole time-of-flight mass spectrometry (LC-Q-TOF-MS) bush tea extract compounds identified before ZnO nanoparticles synthesis using MetFrag software (KEGG and ChemSpider databases, 50 ppm).

	Compound name	Formula	RT [sec]
1	(+)-7-Isojasmonic acid	C _12_H _18_O _3_	294
2	(6Z,9Z,12Z)-Octadecatrienoic acid	C _18_H _30_O _2_	543.6
3	10-Oxo-11,15-phytodienoic acid	C _18_H _28_O _3_	357.6
4	13-hydroxy-9Z,11E-octadecadienoic acid	C _18_H _32_O _3_	652.2
5	17-Hydroxylinolenic acid	C _18_H _30_O _3_	340.8
6	1-O,6-O-Digalloyl-beta-D-glucose (tannin)	C _20_H _20_O _14_	351
7	3,6-Anhydroglucose	C _6_H _10_O _5_	228.6
8	3-hydroperoxy-4-phenyl-pentan-1-ol/Loliolide	C _11_H _16_O _3_	309
9	3-tert-Butyl-5-methylcatechol	C _11_H _16_O _2_	426
10	4-Heptyloxyphenol	C _13_H _20_O _2_	282.6
11	4”-Hydroxyacetophenone	C _8_H _8_O _2_	71.4
12	4-Hydroxyestradiol-17beta	C _18_H _24_O _3_	443.4
13	5,7,3'-Trimethoxy-6,4',5'-trimethoxyisoflavone	C _18_H _16_O _8_	690
14	7-Hydroxy-2”,4”,5”-trimethoxyisoflavone	C _18_H _16_O _6_	726
15	Naringenin 7-O-beta-D-glucoside	C _21_H _22_O _10_	435.6
16	17-Hydroxylinolenic acid	C _18_H _30_O _3_	372.6
17	Adenine	C _5_H _5_N _5_	783
18	alpha-Curcumene	C _15_H _22_	609.6
19	5S-Hydroperoxy-18R-HEPE	C _20_H _30_O _5_	274.8
20	Atropaldehyde	C _9_H _8_O	345
21	Scullcapflavone II	C _19_H _18_O _8_	462.6
22	Cinnamaldehyde	C _9_H _8_O	354
23	Cisapride	C _29_H _27_N _3_O _3_	115.8
24	Coumaric acid/Caffeic Aldehyde	C _9_H _8_O _3_	285
25	Coumarin	C _9_H _6_O _2_	76.8
26	D-Norvaline	C _5_H _11_NO _2_	48
27	Homovanillate/Dihydrocaffeic acid	C _9_H _10_O _4_	288.6
28	Lancerin	C _19_H _18_O _10_	348.6
29	Lophophorine/Stovaine	C _13_H _17_NO _3_	55.8
30	Mallotophenone	C _21_H _24_O _8_	432
31	Malonyldaidzin	C _24_H _22_O _12_	207.6
32	Melampodin A	C _21_H _24_O _9_	399.6
33	Montanol	C _21_H _36_O _4_	513.6
34	Myrcene/(E)-beta-Ocimene	C _10_H _16_	321.6
35	Nafenopin glucuronide	C _26_H _30_O _9_	291
36	Neocnidilide/4-Hexyloxyphenol	C _12_H _18_O _2_	421.8
37	Pentalen-13-ol/Nonylphenol	C _15_H _24_O	411
38	Petasin/Cafestol	C _20_H _28_O _3_	558.6
39	Pinosylvin	C _14_H _12_O _2_	276.6
40	Quinestrol	C _25_H _32_O _2_	232.2
41	Traumatic acid	C _12_H _20_O _4_	403.8
42	Tricin	C _17_H _14_O _7_	379.8
43	Umbelliferone/4-Hydroxycoumarin	C _9_H _6_O _3_	209.4
44	4”-Hydroxyacetophenone	C _8_H _8_O _2_	1.19

Table 2 present the compounds identified from the supernatant after the synthesis of ZnO nanoparticles. The secondary metabolites investigated present a reduced number compared to the ones from the crude extract, thus revealing that a reaction has taken place between bush tea natural extract metabolites and the precursor resulting in the formation of ZnO nanoparticles.

**Table 2.** Liquid chromatography quadrupole time-of-flight mass spectrometry (LC-Q-TOF-MS) bush tea extract compounds identified after ZnO nanoparticles synthesis using MetFrag software (KEGG and ChemSpider, 50 ppm).

	Compound name	Formula	RT [sec]
1	Indanone	C _9_H _8_O	348.6
2	Mallotophenone	C _21_H _24_O _8_	432.6
3	Melampodin A	C _21_H _24_O _9_	399.6
4	Sterigmatocystin	C _18_H _12_O _6_	268.8
5	Umbelliferone	C _9_H _6_O _3_	211.8
6	Salicylate	C _7_H _6_O _3_	182.4
7	Resolvin E2	C _20_H _30_O _4_	265.8
8	Scullcapflavone II	C _19_H _18_O _8_	463.8
9	Myrtenol	C _10_H _16_O	306
10	3-tert-Butyl-5-methylcatechol	C _11_H _16_O _2_	427.8
11	(+)-7-Isojasmonic acid	C _12_H _18_O _3_	404.4
12	Traumatic acid	C _12_H _20_O _4_	91.2
13	4-Heptyloxyphenol	C _13_H _20_O _2_	282.6
14	4,4”-Dihydroxystilbene	C _14_H _12_O _2_	276.6
15	1,3-Diphenylpropane	C _15_H _16_	309.6
16	Geranyl hydroquinone	C _16_H _22_O _2_	781.2
17	Syringin	C _17_H _24_O _9_	232.8
18	3-Hydroxybenzaldehyde	C _7_H _6_O _2_	280.2
19	6-Hydroxyluteolin 7-glucoside	C _21_H _20_O _12_	256.2
20	6-Methoxyaromadendrin 3-O-acetate	C _18_H _16_O _8_	388.8
21	Adenine	C _5_H _5_N _5_	72.6
22	9S-hydroxy-10E,12Z,15Z-octadecatrienoic acid	C _18_H3 _0_O _3_	372.6
23	9E-Heptadecenoic acid	C _17_H _32_O _2_	337.2
24	Carboxymethyloxysuccinate	C _6_H _8_O _7_	81
25	Coumarin	C _9_H _6_O _2_	219
26	Pent-7alpha-Hydroxykaur-16-en-19-oic acid	C _20_H _30_O _3_	319.8
27	Etherolenic acid	C _18_H _28_O _3_	357.6
28	Icariin	C _33_H _40_O _15_	240

Assessment of the implication of bush tea compounds in ZnO nanoparticles synthesis

**Figure 3.**
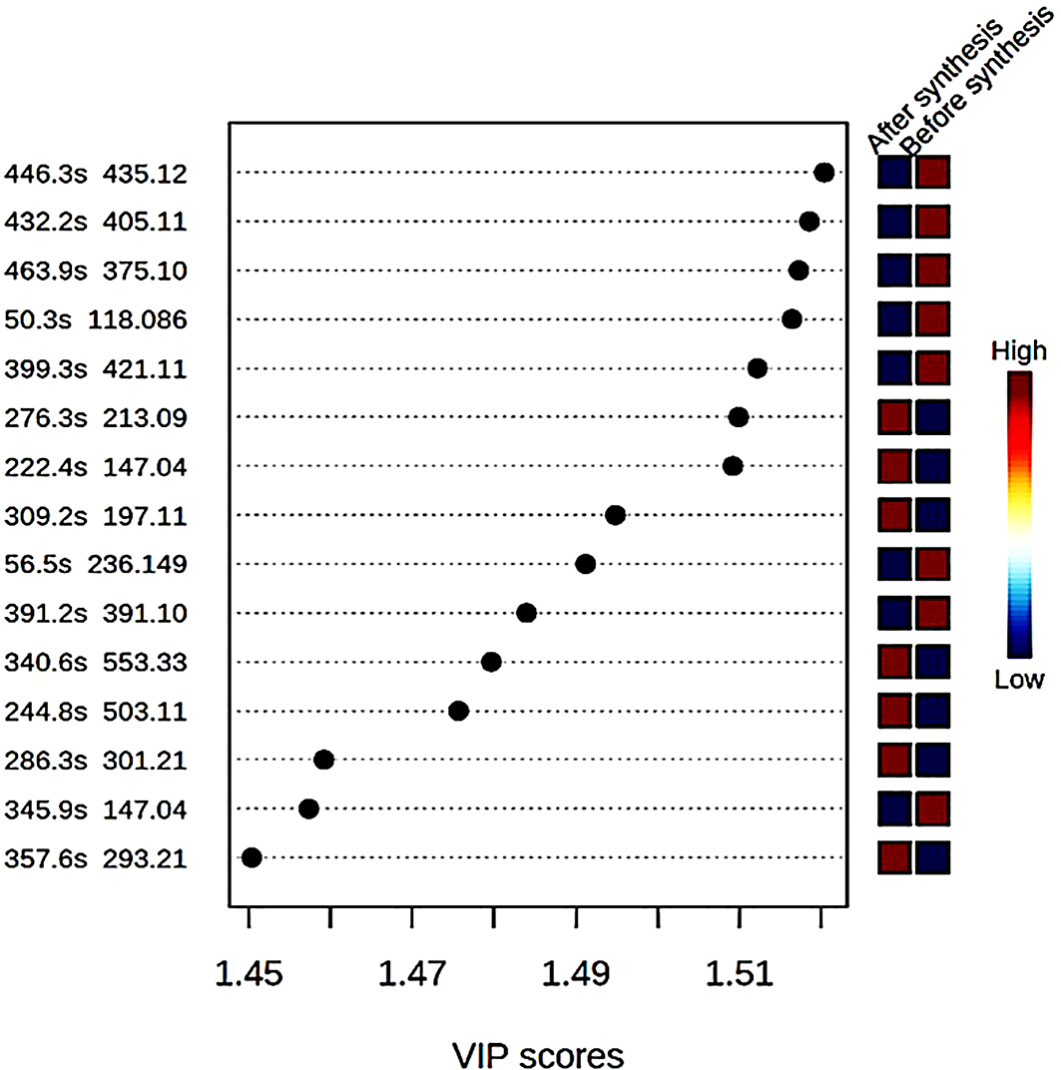
Variable importance in progression (VP) score plot of different compounds found in the bush tea crude extract before synthesis and the supernatant after synthesis of ZnO nanoparticles.

In this study, compound identification was carried out using Bruker data analysis and data profiling tools. The KEGG and ChemSpider databases were consulted to find the name and the chemical formula of each identified compound. The different compounds with mass to ratio (m/z) values as well as their retention time (in seconds) were shown with variable importance in the progression (VIP) score plot ( Figure 3). The concentration of eight compounds was found to be high in the crude extract compared to the supernatant after the synthesis of ZnO nanoparticles where their concentrations were low.

Table 3 present the various compounds that were involved in the synthesis process of ZnO nanoparticles including five flavonoids and two polyphenol compounds, as well as one aromatic compound, which highly reacted with the precursor to form ZnO nanoparticles. Studies have shown that the synthesis of nanoparticles using plant extracts involves terpenoids, flavonoids, alkaloids and phenolic acid, which act as reducing, capping, and stabilizing agents ( Kuppusamy *et al*., 2016).

**Table 3.** Identified compounds reported having mostly interacted with the precursor to form ZnO nanoparticles.

Compound name	Formula	Type
Naringenin 7-O-beta-D-glucoside	C _21_H _22_O _10_	Flavonoid
Scullcapflavone II	C _19_H _18_O _8_	Flavonoid
Mallotophenone	C _21_H _24_O _8_	Polyphenol
6-Methoxyaromadendrin 3-O-acetate	C _18_H _16_O _8_	Flavonoid
2-Phenylacetamide	C _8_H _9_NO	Polyphenol group
7-Hydroxy-2”,4”,5”-trimethoxyisoflavone	C _18_H _16_O _6_	Flavonoid
Coumarin	C _9_H _6_O _2_	Aromatic
Malonyldaidzin	C _24_H _22_O _12_	Flavonoid

### ZnO nanoparticles characterization

XRD analysis

**Figure 4.**
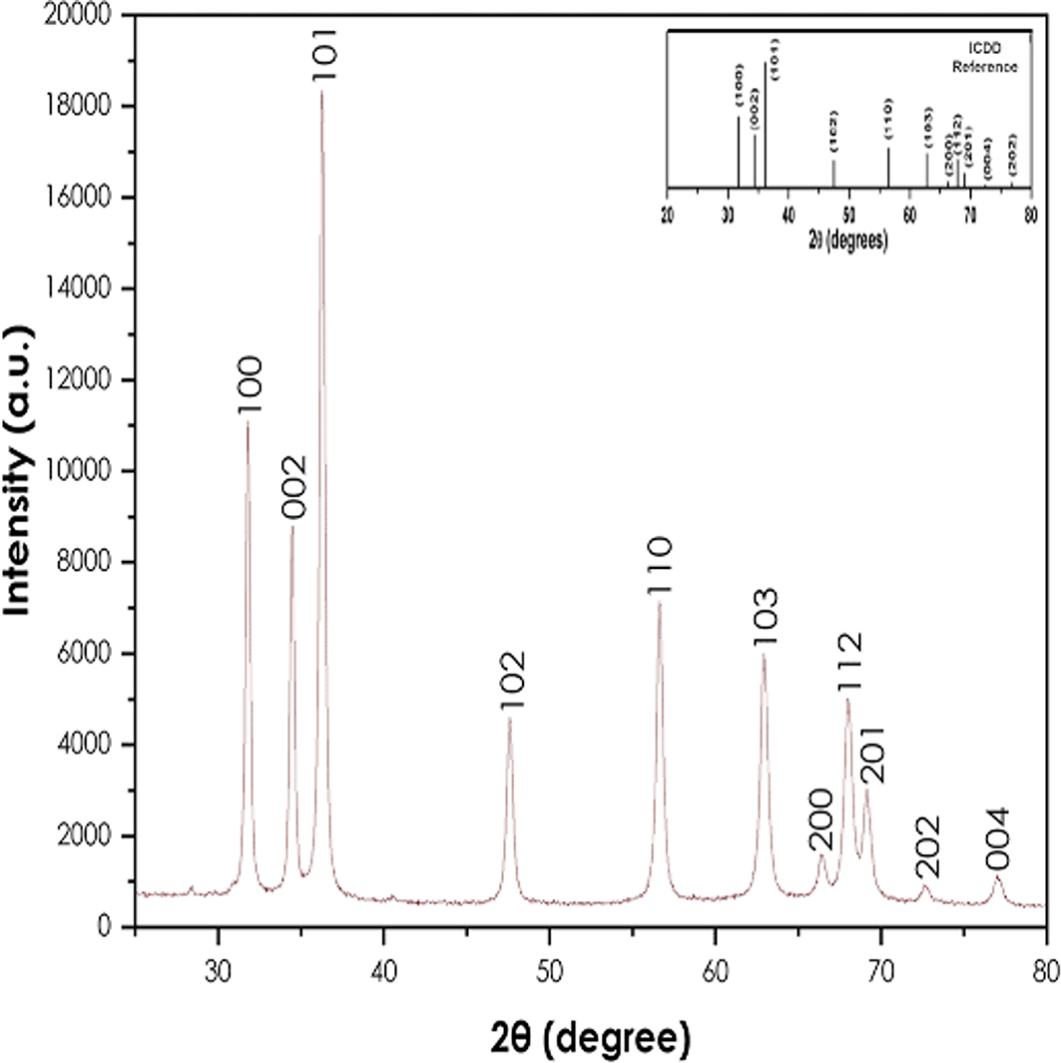
X-ray diffraction pattern of ZnO nanoparticles.

The XRD analysis was done to confirm the crystallinity of the synthesized ZnO nanoparticles using a Bruker AXS (Germany) D8 advance X-ray diffractometer. Figure 4 presents the XRD pattern of the ZnO nanoparticles. The crystallinity of the powder resulting from the synthesis using *A. phylicoides* DC extract. The peaks (100), (002), (101), (102), (110), (103), (200), (112), (201), (004) and (202) are lattice planes. The diffraction peaks reveal that the synthesized ZnO nanoparticles are essentially crystalline, in accord with the ICDD #897102 in the wurtzite structure ( Noman *et al.*, 2020). The same results have been observed by the green synthesis of ZnO nanoparticles using *Ocimum basilicum* ( Salam *et al*., 2014) and *Agathosma betulina* ( Thema *et al*., 2015). The average crystallite size of obtained ZnO nanoparticles calculated using the modified Scherrer equation was approximately 24.53 nm.

Fourier-transform infrared spectroscopy

**Figure 5.**
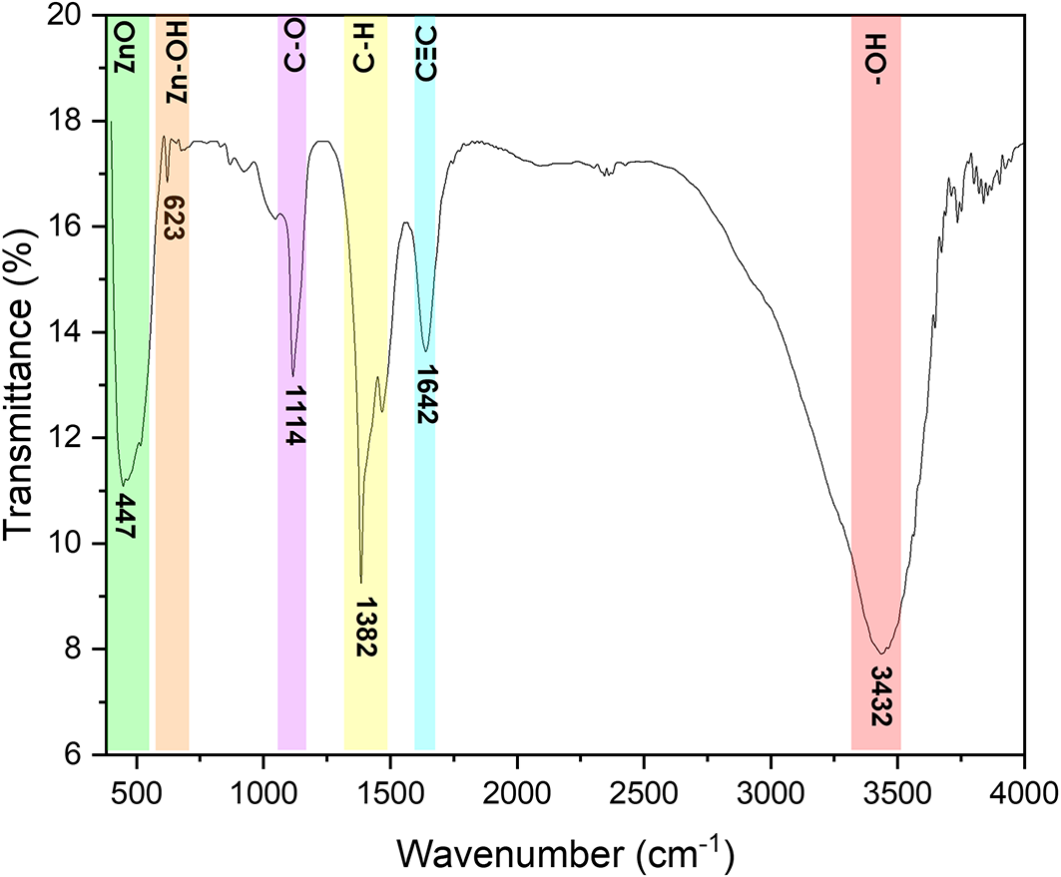
Fourier-transform infrared spectra of ZnO powder annealed at 600°C.

**Figure 6.**
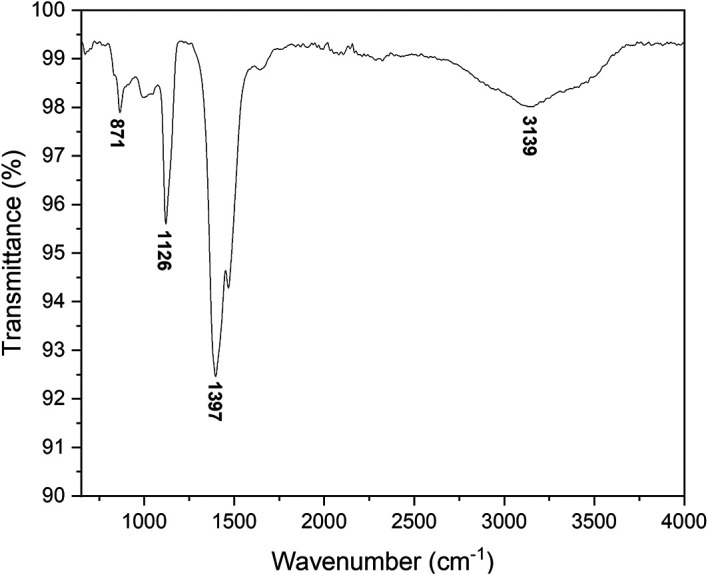
Fourier-transform infrared spectra of Bush tea leaf extract.

The PerkinElmer Frontier FTIR spectrometer was used to perform FTIR analyses using Potassium bromide (KBr) (Potassium bromide) optics. The presence of ZnO nanoparticles was confirmed by the peak at 479 cm ^−1^ as shown in Figure 5. The other observed peaks are attributed to the phytochemical components present in the extract solution. The peak at 1113 cm ^−1^ is attributed to the C-O stretching of primary alcohols. The peak at 1427 cm ^−1^ corresponds to the O-H bending of the carboxylic acid. The peak observed at 2351 cm ^−1^ is attributed to the O=C=O stretching of carbon dioxide. The FTIR spectra of bush tea extract, presented in Figure 6, show the presence of carboxylic acid bonding, primary alcohol stretching as well as the intramolecular hydrogen bond.

UV-Vis analysis

**Figure 7.**
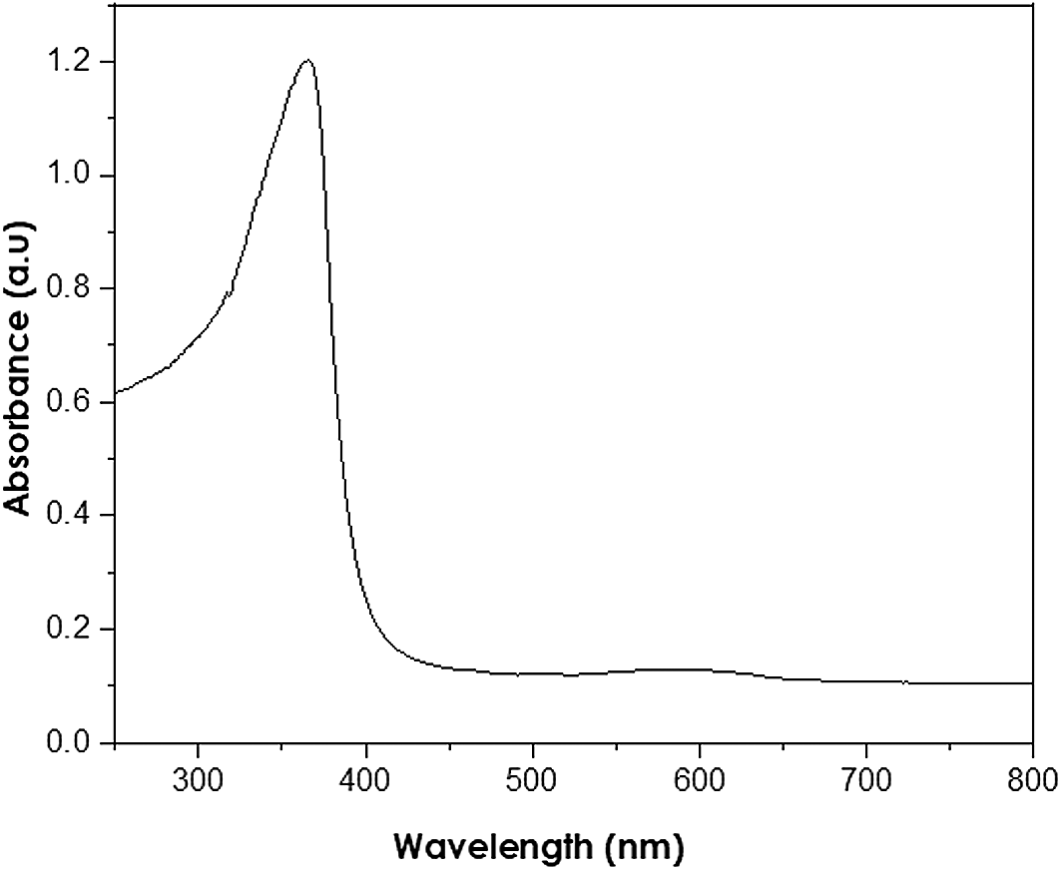
Ultraviolet-visible spectra of as-synthesized ZnO nanoparticles.

UV-Vis analyses were performed at a resolution of 1 nm at a 250–800 nm wavelength range using a PerkinElmer Lambda 650S UV-Vis spectrometer. The absorption of ZnO nanoparticles is observed in the wavelength range of 250–400 nm ( Kolekar *et al*., 2011). The measured peak at 380 nm (as shown in Figure 7) reveals the presence of ZnO nanoparticles with a band gap energy of 3.11 eV, smaller than the bulk ZnO of 3.37 eV. Thus, the presence of hexagonal wurtzite structures in the analysed samples is indicated, in accordance with the XRD results.

SEM and EDS analyses

**Figure 8.**
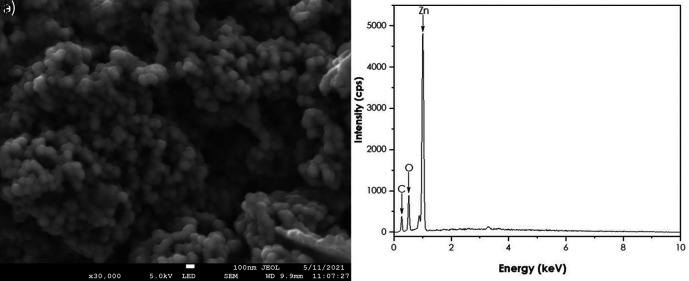
(a) Scanning electron microscopy image and (b) Energy-dispersive X-ray spectra of ZnO nanoparticles.

A JEOL JSM-7500F field-emission scanning electron microscope (FE-SEM) coupled with a JXA-8230/SXEDS/EDS/WDS energy-dispersive X-ray spectrometer (EDS) was used to get the morphology and the purity of the ZnO nanoparticles. SEM results are represented in Figure 8. The image shows quasi-spherical shaped ZnO nanoparticles agglomerated together. The EDS confirmed the presence of Zn and O. These findings are supported by Nethavhanani (2017) using natural extracts of *Aspalathus linearis* as a reducing agent ( Nethavhanani, 2017).

## Discussion

Understanding the process of nanoparticle synthesis using the green route is key to the efficiency of the process and the outcome. Following the lack of data on chemical interactions of plant extracts with different metals to form nanoparticles, this study aimed to investigate the interaction of compounds with zinc nitrate to form ZnO nanoparticles. The identification of plant metabolites was performed using LC-MS tools employing different databases such as KEGG, ChemSpider or Metfrag ( Cecilia *et al*., 2020). Henceforth, the differences in the extracts resulting from the synthesis of ZnO nanoparticles were shown utilizing PCA and the VIP score plot. Bush tea leaves contain a high percentage of flavonoids and tannins, apart from non-structural carbohydrates, proteins, fatty acids, and minerals, such as calcium, magnesium, phosphorus, potassium, sodium, iron, manganese, zinc, copper, aluminium, sulphur and fluoride ( Lerotholi *et al.*, 2017). Hence, the synthesis process resulted in the complete use of some metabolites as shown in Figure 2. The supernatant recorded low quantities of 8-C-Glucosylnaringenin/Naringenin 7-O-beta-D-glucoside, Scullcapflavone II, Mallotophenone, 6-Methoxyaromadendrin 3-O-acetate, 2-Phenylacetamide, 7-Hydroxy-2″,4″,5″-trimethoxyisoflavone, Coumarin, Malonyldaidzin ( Figure 3). A variety of metabolites, such as terpenoids, polyphenols, sugars, alkaloids, phenolic acids, and proteins can reduce metal ions into nanoparticles ( Marslin *et al*., 2018). Flavonoids, polyphenols as well as an aromatic compound interacted most with the precursor to form ZnO nanoparticles ( Table 2). UV-Vis is a wonderful tool for the examination of the size and the shape of nanoparticles ( Raut Rajesh *et al*., 2009). The analysed samples show the presence of a wurtzite structure at 380 nm. These findings are supported by ( Krupa and Vimala, 2016) who reported the synthesis of ZnO nanoparticles absorbing light at 368 nm. The wavelength of 380 nm corresponds to the bulk band-edge of 3.2 eV for ZnO ( Kolekar *et al*., 2011).

## Conclusion

In this study, bush tea metabolites were screened to understand their interaction with metal ions to form nanoparticles. The LC-MMS peaks in both the crude extract before ZnO nanoparticles synthesis and the supernatant after synthesis revealed a significant difference, shown by the PCAs. Different flavonoids, polyphenols and an aromatic compound were found to react with zinc nitrate to form zinc nanoparticles. The FTIR as well as the XRD and UV-Vis analyses confirmed the formation of ZnO nanoparticles with a hexagonal wurtzite structure.

## Data availability

All data underlying the results are available as part of the article and no additional source data are required.
