# Supplementary material for: Green synthesis and characterization of zinc oxide nanoparticles using bush tea ( Athrixia phylicoides DC) natural extract: assessment of the synthesis process
Source: F1000Res. 2022 Aug 23;10:1077. Originally published 2021 Oct 25. [Version 4] doi: 10.12688/f1000research.73272.4 (PMC9520229; doi:10.12688/f1000research.73272.4)
Supplement: Peer review history

# Peer review: Reviewer response for version 4

**DOI:** 10.5256/f1000research.137542.r148377

**Published:** 2022-10-03

**Authors:** Karina Chávez

**Affiliations:** 1 Instituto de Investigación en Metalurgia y Materiales, Universidad Michoacana de San Nicolás de Hidalgo, UMSNH, Morelia, Mexico

The requested corrections were attended.

Is the work clearly and accurately presented and does it cite the current literature?

Partly

If applicable, is the statistical analysis and its interpretation appropriate?

Partly

Are all the source data underlying the results available to ensure full reproducibility?

Partly

Is the study design appropriate and is the work technically sound?

Partly

Are the conclusions drawn adequately supported by the results?

Yes

Are sufficient details of methods and analysis provided to allow replication by others?

No

Reviewer Expertise:

Green synthesis and materials characterization

I confirm that I have read this submission and believe that I have an appropriate level of expertise to confirm that it is of an acceptable scientific standard.

# Peer review: Reviewer response for version 4

**DOI:** 10.5256/f1000research.137542.r150557

**Published:** 2022-09-28

**Authors:** Hanady S. Al-Shmgani

**Affiliations:** 1 Biology Department, College of Education for Pure Science/Ibn al-Haitham, University of Baghdad, Baghdad, Iraq

This manuscript describes the biosynthesis of zinc oxide nanoparticles from bush tea ( *Athrixia phylicoides* DC), the original aim of that work is interesting to read and should be considered for indexing. I believe the authors have adequately responded to the previous reviewers' suggestions in this revised version of the manuscript. Only one minor revision, the Conclusions seems to be repeat of results. It should highlight the insights and applicability of your findings/results for future work, rather than simply summarizing the key findings of the study.

Is the work clearly and accurately presented and does it cite the current literature?

Yes

If applicable, is the statistical analysis and its interpretation appropriate?

Not applicable

Are all the source data underlying the results available to ensure full reproducibility?

Yes

Is the study design appropriate and is the work technically sound?

Yes

Are the conclusions drawn adequately supported by the results?

Partly

Are sufficient details of methods and analysis provided to allow replication by others?

Yes

Reviewer Expertise:

Green-NPs synthesis, Cancer research, antioxidant

I confirm that I have read this submission and believe that I have an appropriate level of expertise to confirm that it is of an acceptable scientific standard.

# Peer review: Reviewer response for version 3

**DOI:** 10.5256/f1000research.125894.r145453

**Published:** 2022-08-12

**Authors:** Karina Chávez

**Affiliations:** 1 Instituto de Investigación en Metalurgia y Materiales, Universidad Michoacana de San Nicolás de Hidalgo, UMSNH, Morelia, Mexico

The manuscript discusses about studying the zinc oxide nanoparticles by a green method using the bush tea. But there are some important major notes about this manuscript:

1. Check for grammatical error, typo error, etc. (especially in the introduction section).
2. If possible, the authors should present a scheme for the method using the Bush tea as a reducing agent, which has advantages or differences from other techniques. Otherwise, it is not easy for us to get the novelty.
3. For the XRD results, I strong suggest that the standard pattern of ZnO should be added in Figure 4, then the figure will more readable to readers.
4. The details of the FTIR results of figures 5 and 6 should be described in the figure, such as the compounds or the wavenumber.
5. The scale of the SEM image (μm) is not comparable for nanoparticles. Add images with higher magnifications or add a TEM analysis. Also, the scale marker is small and not appropriate, I suggest editing it.

Is the work clearly and accurately presented and does it cite the current literature?

Partly

If applicable, is the statistical analysis and its interpretation appropriate?

Partly

Are all the source data underlying the results available to ensure full reproducibility?

Partly

Is the study design appropriate and is the work technically sound?

Partly

Are the conclusions drawn adequately supported by the results?

Yes

Are sufficient details of methods and analysis provided to allow replication by others?

No

Reviewer Expertise:

Green synthesis and materials characterization

I confirm that I have read this submission and believe that I have an appropriate level of expertise to confirm that it is of an acceptable scientific standard, however I have significant reservations, as outlined above.

# Peer review: Reviewer response for version 3

**DOI:** 10.5256/f1000research.125894.r134572

**Published:** 2022-05-11

**Authors:** Saeid Taghavi Fardood

**Affiliations:** 1 Department of Chemistry, Faculty of Science, Ilam University, Ilam, Iran

The manuscript is appropriate for indexing.

Is the work clearly and accurately presented and does it cite the current literature?

Partly

If applicable, is the statistical analysis and its interpretation appropriate?

Partly

Are all the source data underlying the results available to ensure full reproducibility?

Partly

Is the study design appropriate and is the work technically sound?

Partly

Are the conclusions drawn adequately supported by the results?

Yes

Are sufficient details of methods and analysis provided to allow replication by others?

Partly

Reviewer Expertise:

Nanoparticles; green synthesis, photocatalysis, dye degradation; metals oxide.

I confirm that I have read this submission and believe that I have an appropriate level of expertise to confirm that it is of an acceptable scientific standard.

# Peer review: Reviewer response for version 2

**DOI:** 10.5256/f1000research.80148.r119826

**Published:** 2022-02-17

**Authors:** Saeid Taghavi Fardood

**Affiliations:** 1 Department of Chemistry, Faculty of Science, Ilam University, Ilam, Iran

The manuscript needs a careful major revision, before indexing **.** The comments are given below.

 1. Synthesis of zinc oxide nanoparticles has been synthesized using other plant extracts. It is better for the authors to compare the present nanoparticles with previous works.

 2. Introduction section should be improved and updated, such as by following these references of mine:

- Green synthesis of zinc oxide nanoparticles using arabic gum and photocatalytic degradation of direct blue 129 dye under visible light ^1^.
- Sol-gel Synthesis and Characterization of Zinc Oxide Nanoparticles Using Black Tea Extract ^2^.
- Green Synthesis of ZnO Nanoparticles via Sol-gel Method and Investigation of Its Application in Solvent-free Synthesis of 12-Aryl-tetrahydrobenzo[α]xanthene-11-one Derivatives Under Microwave Irradiation ^3^.
- Facile green synthesis and characterization of zinc oxide nanoparticles using tragacanth gel: investigation of their photocatalytic performance for dye degradation under visible light irradiation ^4^.
- Magnetic Mg _0.5_Zn _0.5_FeMnO _4_ nanoparticles: Green sol-gel synthesis, characterization, and photocatalytic applications ^5^.
- Facile green synthesis, characterization and visible light photocatalytic activity of MgFe _2_O _4_@CoCr _2_O _4_ magnetic nanocomposite ^6^.

In the proposed references, in all cases, nanoparticles have been synthesized using green synthesis. In the first 4 references, the green synthesis of ZnO nanoparticles has been studied. The authors should express their work with this method of synthesis in terms of size and quality and express their superiority. In the next two references, the green synthesized nanoparticles have been studied using advanced methods and their application as a suitable photocatalyst has been studied, which can help the authors and readers of the article in how to study their analysis and interpretation.

 3. For better comparison of synthesized sample, determination of specific surface area, pore diameter and total pore volume of samples, BET analysis is advised.

 4. SEM image scale (μm) is not suitable for nanoparticles. It is better to add image scale around (500 and 200 nm) and high quality. If possible, TEM analysis should be performed.

 5. It would have been better if the authors had studied the catalytic, photocatalytic, or biological applications of synthesized nanoparticles. What is the authors' goal of synthesizing nanoparticles without examining their application?

Is the work clearly and accurately presented and does it cite the current literature?

Partly

If applicable, is the statistical analysis and its interpretation appropriate?

Partly

Are all the source data underlying the results available to ensure full reproducibility?

Partly

Is the study design appropriate and is the work technically sound?

Partly

Are the conclusions drawn adequately supported by the results?

Yes

Are sufficient details of methods and analysis provided to allow replication by others?

Partly

Reviewer Expertise:

Nanoparticles; green synthesis, photocatalysis, dye degradation; metals oxide.

I confirm that I have read this submission and believe that I have an appropriate level of expertise to confirm that it is of an acceptable scientific standard, however I have significant reservations, as outlined above.

## References

[1] : Green synthesis of zinc oxide nanoparticles using arabic gum and photocatalytic degradation of direct blue 129 dye under visible light. *Journal of Materials Science: Materials in Electronics* .2017;28(18) : 10.1007/s10854-017-7199-5 13596-13601 10.1007/s10854-017-7199-5

[2] : Sol-gel Synthesis and Characterization of Zinc Oxide Nanoparticles Using Black Tea Extract. *Journal of Applied Chemical reseacrh* .2017;11(4) :

[3] : Green Synthesis of ZnO Nanoparticles via Sol-gel Method and Investigation of Its Application in Solvent-free Synthesis of 12-Aryl-tetrahydrobenzo[α]xanthene-11-one Derivatives Under Microwave Irradiation. *Chemical Methodologies* .2019;3(6) : 10.33945/SAMI/CHEMM.2019.6.2 10.33945/SAMI/CHEMM.2019.6.2

[4] : Facile green synthesis and characterization of zinc oxide nanoparticles using tragacanth gel: investigation of their photocatalytic performance for dye degradation under visible light irradiation. *Nanochemistry Research* .2020;5(1) : 10.22036/NCR.2020.01.007 10.22036/NCR.2020.01.007

[5] : Magnetic Mg0.5Zn0.5FeMnO4 nanoparticles: Green sol-gel synthesis, characterization, and photocatalytic applications. *Journal of Cleaner Production* .2021;288: 10.1016/j.jclepro.2020.125632 10.1016/j.jclepro.2020.125632

[6] : Facile green synthesis, characterization and visible light photocatalytic activity of MgFe2O4@CoCr2O4 magnetic nanocomposite. *Journal of Photochemistry and Photobiology A: Chemistry* .2022;423: 10.1016/j.jphotochem.2021.113621 10.1016/j.jphotochem.2021.113621

# Response

**Published:** 2022-04-07

**Authors:** Gabriel Amani Kaningini

Dear Dr Fardood,

 Thank you very much for the comments that have helped us improve our article.

 Please see below the response to the comments:

1. The work has been compared to other research in the discussion section.
2. Introduction section amended.
3. BET analysis not done due to the kind of study that was led (ref point 5).
4. Image added at 100 nm scale.
5. The work is mainly to explain the dynamics of compounds involved in the synthesis process of ZnO nanoparticles. We have reported the end product to show that ZnO NPs were synthesized.

Please let us know of any concern.

 Kindly

 AG Kaningini

# Peer review: Reviewer response for version 1

**DOI:** 10.5256/f1000research.76914.r100333

**Published:** 2021-11-24

**Authors:** Mohammod Aminuzzaman

**Affiliations:** 1 Department of Chemical Science, Faculty of Science, Perak Campus, Jalan Universiti, Universiti Tunku Abdul Rahman (UTAR), Kampar, Malaysia

The authors reported on the “Green synthesis and characterization of zinc oxide nanoparticles using bush tea ( *Athrixia phylicoides* DC) natural extract: assessment of the synthesis process”. This work is of interest and of certain significance for chemical processing. But some major modifications are needed before being accepted for indexing:

1. Importantly, there are no experiments discussing the practical utilization of the so-produced ZnO NPs. For example, photocatalytic activities under UV-vis-light irradiation or biological activities. Please include one application of the synthesized ZnO NPs.
2. TEM image of ZnO NPs must be included in the manuscript.
3. Band gap energy of ZnO NPs must be calculated using Tauc Plot.
4. FT-IR spectra of the leaves of bush tea ( *A. phylicoides* DC) should be be included in the manuscript.

Is the work clearly and accurately presented and does it cite the current literature?

Partly

If applicable, is the statistical analysis and its interpretation appropriate?

Not applicable

Are all the source data underlying the results available to ensure full reproducibility?

Yes

Is the study design appropriate and is the work technically sound?

Partly

Are the conclusions drawn adequately supported by the results?

Yes

Are sufficient details of methods and analysis provided to allow replication by others?

Partly

Reviewer Expertise:

Green synthesis of Nanomaterials, Solar cells, Photocatalyst

I confirm that I have read this submission and believe that I have an appropriate level of expertise to state that I do not consider it to be of an acceptable scientific standard, for reasons outlined above.

# Response

**Published:** 2021-11-26

**Authors:** Gabriel Amani Kaningini

Dear Aminuzzaman,

 Thank you for the comments on our manuscript. I would like to highlight some particulars regarding the work we have done according to the comments:

1. The study was to confirm the ability of bush tea to synthesize ZnO nanoparticles as the title indicates “assessment of the synthesis process”. This is the reason why we explain and highlight the different compounds involved in the synthesis process, the ZnO nanoparticles being the end product of our work we haven’t tested it for any practical use. However, further work is undergoing for their use as fertilizers or growth-promoting agents.
2. We would really like to add the TEM images, however, we have found ourselves limited to the characterization techniques that we have cited in the work. I do believe that the SEM images of the nanoparticles are enough.
3. We have worked on the bandgap energy (UV-Vis results).
4. We have added the FTIR results of the bush tea leaves extract as recommended.
